# Effect of Clay Minerals on Carbonate Precipitation Induced by Cyanobacterium *Synechococcus* sp.

**DOI:** 10.1128/spectrum.00363-23

**Published:** 2023-04-11

**Authors:** Xiao Wang, Xiangxin Kong, Qian Liu, Kun Li, Zaixing Jiang, Hengjun Gai, Meng Xiao

**Affiliations:** a State Key Laboratory Base for Eco-Chemical Engineering in College of Chemical Engineering, Qingdao University of Science and Technology, Qingdao, China; b School of Energy Resources, China University of Geosciences (Beijing), Beijing, China; Connecticut Agricultural Experiment Station

**Keywords:** clay minerals, aggregation, *Synechococcus*, calcite, aragonite

## Abstract

Carbonate precipitation induced by cyanobacteria is an important factor in lacustrine fine-grained carbonate rock genesis. As key components of these rocks, clay minerals play an important role in aggregating cyanobacteria. However, the formation mechanism of fine-grained carbonate under the effect of clay minerals is unclear. In this study, we investigated carbonate precipitation by *Synechococcus* cells under the influence of clay minerals. The results showed that clay minerals can accelerate *Synechococcus* aggregation, and the aggregation rate of the kaolinite group was significantly higher than that of montmorillonite. The aggregate size and *Synechococcus* cell content increased with an increase in clay minerals, resulting in increasing organic matter and carboxyl content in the aggregates. Due to the high affinity between carboxyl and Ca^2+^, the presence of *Synechococcus* sp. could improve the Mg/Ca molar ratio in the microenvironment of aggregates, which is conducive to aragonite precipitation. Thus, aragonite 5 to 10 μm in size precipitated when *Synechococcus* and clay minerals coexisted, whereas low-magnesium calcite (15 to 60 μm) was the main carbonate only in the presence of *Synechococcus*. This study provides important insights into the mechanisms of microbial-induced carbonate precipitation under the effect of clay minerals, which might offer theoretical support for the genesis of fine-grained lacustrine carbonate.

**IMPORTANCE** The biogenesis of lacustrine fine-grained carbonates is of great significance to the exploitation of shale oil. Clay minerals are an important component of lacustrine fine-grained sedimentary rocks, which is conductive to the aggregation and settlement of cyanobacteria. We investigated the precipitation of carbonate induced by *Synechococcus* sp. with the addition of kaolinite and montmorillonite. The pH and calcium carbonate saturation of the environment increased under the effect of cyanobacteria photosynthesis. The aggregation of cyanobacteria cells increased the Mg/Ca molar ratio of the microenvironment, creating a favorable condition for the precipitation of aragonite, which was similar in size to the micritic calcite of fine-grained sedimentary rocks. This study provides theoretical support for the genesis of fine-grained carbonates.

## INTRODUCTION

In recent years, with the worldwide development of shale oil and gas exploration, studies on lacustrine fine-grained sedimentary rocks have received unprecedented attention (1, 2). Carbonate-rich, fine-grained sedimentary rocks are important carriers of lacustrine shale oil and understanding their genesis is important for the development of lacustrine carbonate-rich oil and gas (3). Geologists have considered that calcite, especially micritic calcite (5 to 20 μm), is a biogenic source because of its relatively high carbon isotope value (C-isotope value, δ^13^C) in fine-grained sedimentary rocks (3, 4). Furthermore, type I and type II kerogen are commonly found in these rocks, indicating that this organic matter might be derived from phytoplankton (such as algae or cyanobacteria) (5, 6). Plankton-derived organic matter is an important source of hydrocarbons in fine-grained sedimentary rock.

Microbial-induced carbonate precipitation (MICP) is a natural phenomenon which is governed by four key factors (7, 8): appropriate Ca^2+^ concentration, dissolved inorganic carbon (DIC) concentration, appropriate pH value, and nucleation site availability. Carbonate precipitation can be induced during the growth and metabolism of many types of microorganisms, such as algae, bacteria, and archaea. For instance, cyanobacteria of the genus *Synechococcus* are important primary producers that can induce carbonate precipitation (9). As early as 1911, it was reported that some microbes could produce calcium carbonate in the ocean (10). J. B. Thompson and F. G. Ferris (11) utilized lake water to culture *Synechococcus* sp. and observed the precipitation of calcite on the cell surface. Additionally, it has been confirmed that cyanobacteria play an important role in calcium carbonate precipitation in microbial mats (12, 13). The negatively charged extracellular polymers on the surface of cyanobacteria can not only adsorb Ca^2+^ and Mg^2+^ but also serve as nucleation sites for mineral precipitation (14). The pH value and CaCO_3_ saturation of the surrounding microenvironment can be improved through cyanobacteria photosynthesis. Carbonic anhydrase (CA) of cyanobacteria catalyzes the utilization of HCO_3_^–^ and accelerates the precipitation of CaCO_3_ (15, 16). Carbonate minerals induced by microbes always have a wide particle size distribution and poor crystallization. Amorphous calcium carbonate (ACC) is the most thermodynamically unstable calcium carbonate and is formed during the early stages of MICP (17). ACC has low solubility and is usually transformed into other stable calcium carbonates, such as calcite, vaterite, or aragonite (18).

Cyanobacteria are important sources of organic matter in lacustrine and marine sediments. However, cyanobacteria cells are small, and it is difficult for them to settle and aggregate freely. Even if they settle at the bottom of the water body, they are easily decomposed by heterotrophic microbes (19). Therefore, the preservation of phytoplankton as organic matter requires the involvement of other factors. Clay minerals, as the key material source and component of sedimentary rocks, have an effect on aggregating cyanobacteria cells, and the settling velocity of cyanobacteria cells can be improved after aggregation (20). The volume and density of aggregate particles are affected by the cation exchange capacity of clay minerals. Using kaolinite and bentonite as examples, bentonite is better than kaolinite at aggregating cyanobacteria cells (19). Clay minerals can aggregate cyanobacteria to form aggregations approximately 10 to 100 μm in diameter and the settling rate increases with increasing particle size, ensuring that the aggregates can pass through the water body and enter into the sediments to quickly avoid being preyed upon (19) and that the aggregates as organic matter can be preserved effectively in an anaerobic lake-bottom environment (21). Thus, clay minerals play an important role in the accumulation and settling of organic matter.

Although the phenomenon of MICP has been simulated and confirmed by laboratory experiments, the influence of clay minerals has rarely been considered. As important components of fine-grained sedimentary rocks, clay minerals strongly aggregate cyanobacteria. However, whether the precipitated carbonate is affected by clay minerals is unknown. In this study, the MICP process was simulated using *Synechococcus* sp. under the influence of clay minerals. Here, we investigated the morphological characteristics of the precipitates and discuss the formation mechanism of carbonates. We focused on the following two aspects: (i) the composition and morphology of carbonate minerals and (ii) the effect of clay minerals on the MICP process.

## RESULTS

### Aggregation of *Synechococcus* sp.

**FIG 1:**
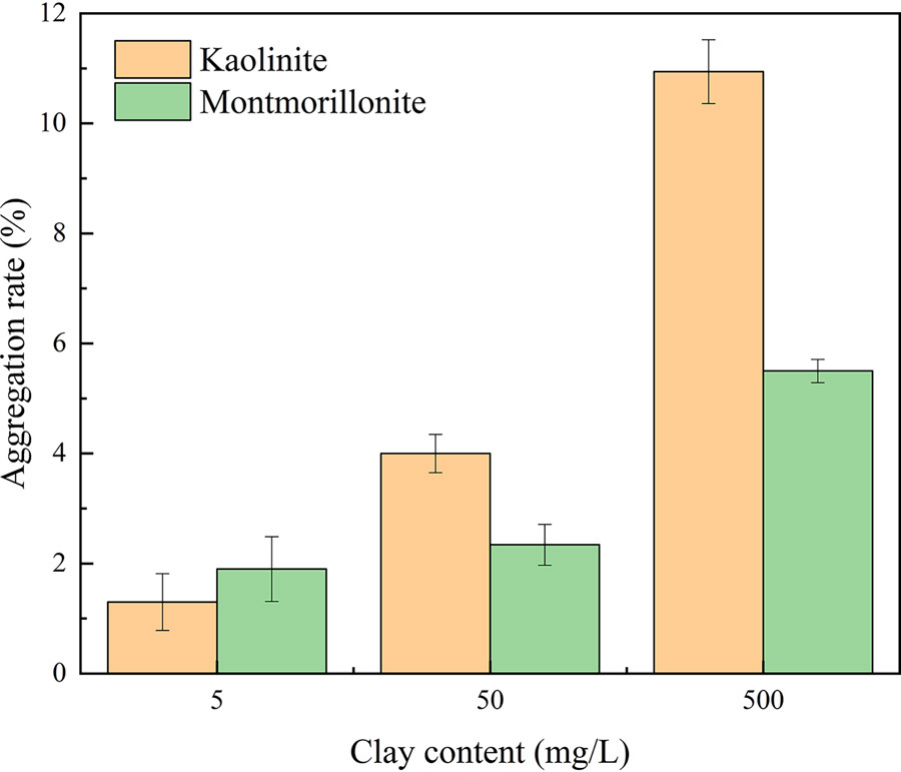
Aggregation rate of *Synechococcus* cells by clay minerals at different concentrations.

Aggregation experiments were conducted by inoculating clay minerals inoculated with cyanobacteria for 2 h, and the results showed that the aggregation rate increases with increasing clay mineral content (Fig. 1). The highest aggregation rates were 10.94% and 5.5% for kaolinite and montmorillonite, respectively, and the aggregation rate of kaolinite was significantly higher than that of montmorillonite, indicating that clay minerals have a significant effect on cyanobacteria aggregation.

### Mg/Ca molar ratio.

**FIG 2:**
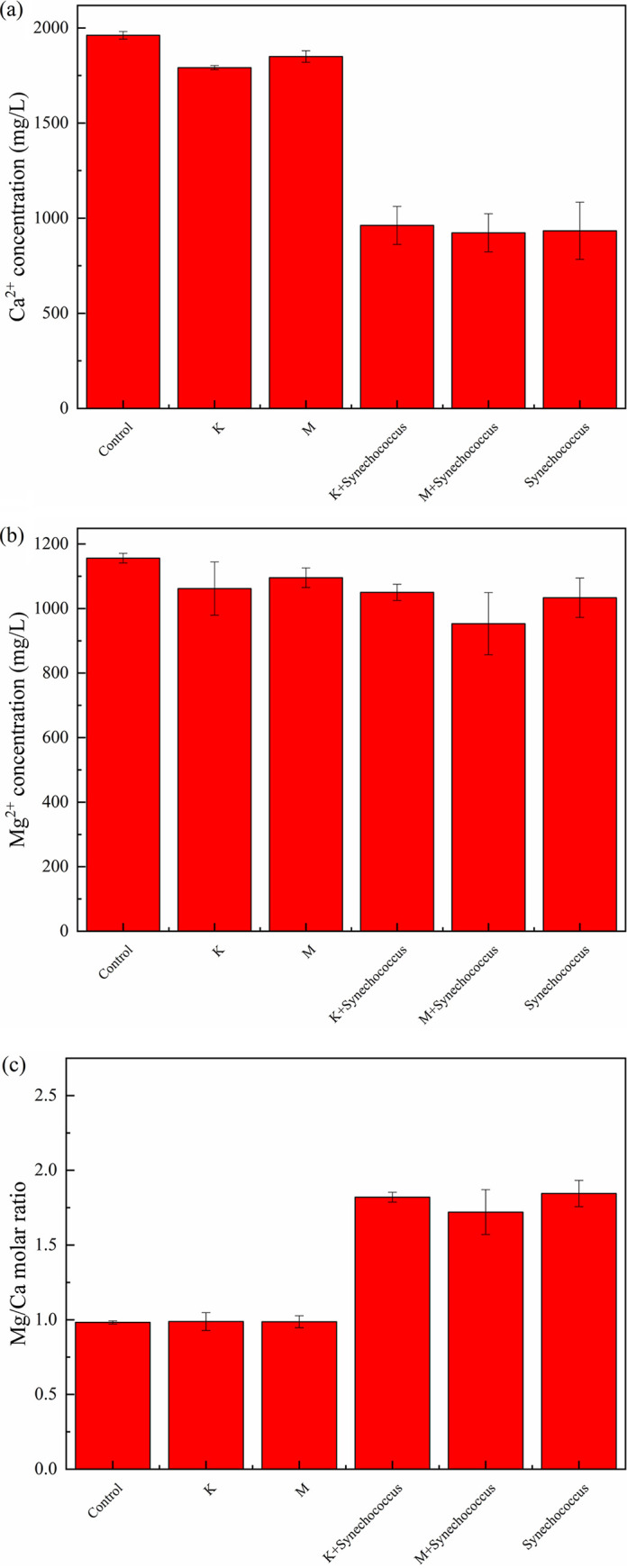
Changes in Ca^2+^/Mg^2+^ concentration and the Mg/Ca molar ratio under the effect of clay minerals. (a to c) Concentration of Ca^2+^ (a), concentration of Mg^2+^ (b), and Mg/Ca molar ratio (c). M, montmorillonite; K, kaolinite.

Carbonate-inducing experiments were conducted for 12 d, and samples with clay mineral concentrations of 0 and 50 mg/L were selected as representatives to investigate the Mg/Ca molar ratio. As shown in Fig. 2, the concentrations of Ca^2+^ were 1,961.13, 1,791.18, and 1,849.85 mg/L for the control, kaolinite, and montmorillonite groups, respectively. The adsorption rates of Ca^2+^ were 8.67% and 5.67% for the kaolinite and montmorillonite groups, respectively. The adsorption rates of Mg^2+^ were 8.15% and 5.23% for the kaolinite and montmorillonite groups, respectively. Thus, there were few differences in the adsorption rates of Ca^2+^ and Mg^2+^ between the kaolinite and montmorillonite groups, although the adsorption rate of kaolinite was slightly higher than that of montmorillonite. As shown in Table S1 in the supplemental material, Ca^2+^ and Mg^2+^ could be leached from clay minerals and their concentrations increased with clay content. However, the concentrations of Ca^2+^ and Mg^2+^ leached from clay were far lower than those in BG11 medium. Thus, there was little influence on the experimental results. When *Synechococcus* sp. was added to the culture medium, the pH increased (Fig. S1) and the concentrations of Ca^2+^ and Mg^2+^ decreased significantly. Taking Ca^2+^ as an example (Fig. 2A), when only *Synechococcus* sp. was present in the solution, the Ca^2+^ concentration exhibited the greatest reduction (52.38%). When clay minerals and *Synechococcus* coexisted, the Ca^2+^ concentration decreased by approximately 42.28% and 47.25% for kaolinite and montmorillonite, respectively. The Mg/Ca molar ratio also changed significantly. As shown in Fig. 2C, the Mg/Ca molar ratios were 0.9826, 0.9882, 0.9872, 1.8198, 1.7206, and 1.8448 for the control, kaolinite, montmorillonite, kaolinite + *Synechococcus*, montmorillonite + *Synechococcus*, and *Synechococcus* groups, respectively, indicating that the presence of *Synechococcus* sp. significantly increased the Mg/Ca molar ratio. The Mg/Ca molar ratio in the presence of clay minerals and *Synechococcus* sp. was slightly lower than that in the presence of *Synechococcus* sp. alone.

### X-ray diffraction.

**FIG 3:**
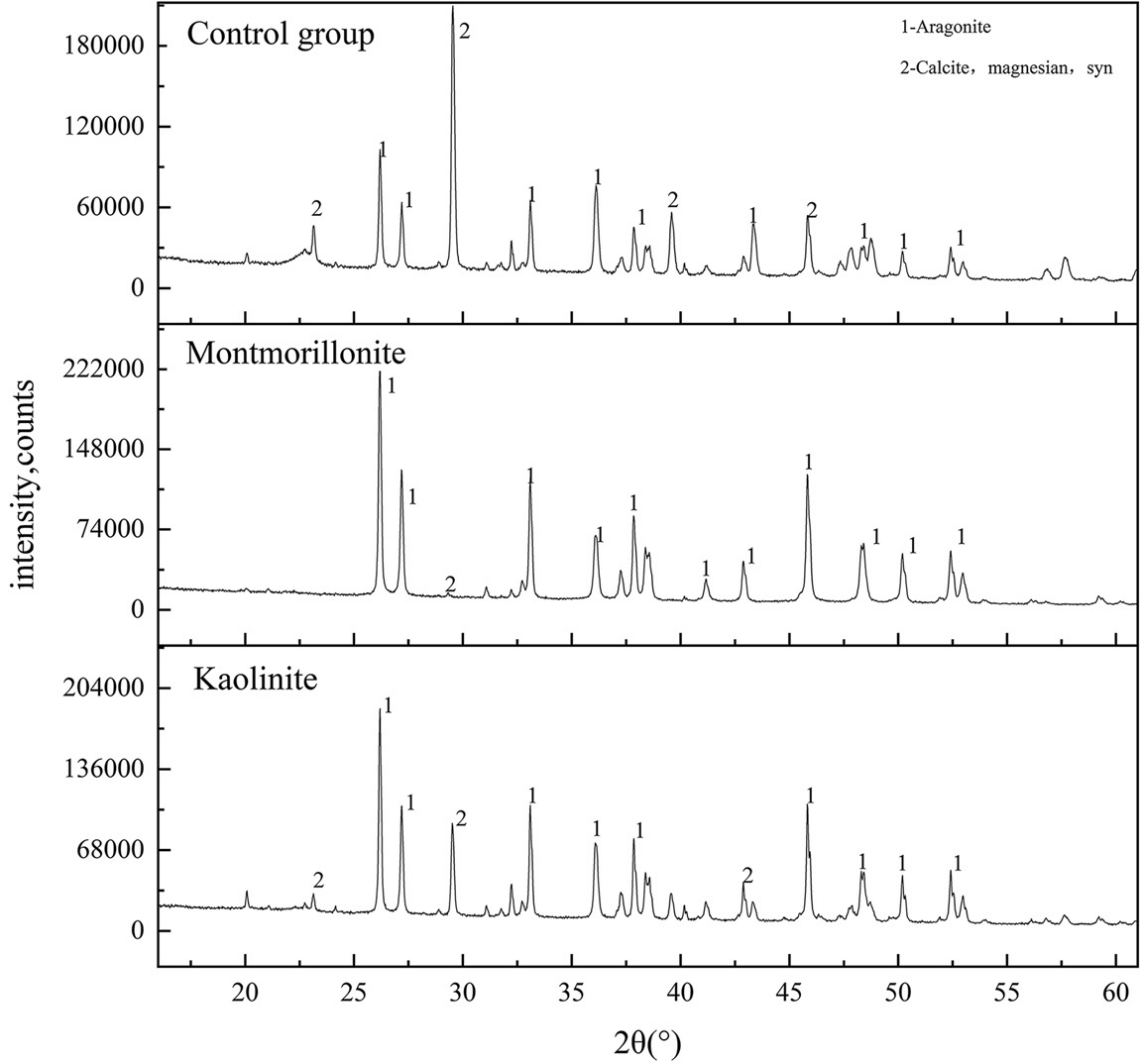
X-ray diffraction patterns of sediments for different samples.

After the carbonate-inducing experiments, precipitates were obtained and purified. The X-ray diffraction (XRD) patterns of the precipitates are shown in Fig. 3. The minerals in all groups were aragonite and low-magnesium calcite. The minerals induced by *Synechococcus* sp. were mainly aragonite and low-magnesium calcite. The 2θ positions of aragonite diffraction peak were 26.2°, 27.2°, 33.1°, 36.1°, 37.9°, 41.2°, 42.9°, 45.9°, 48.3°, 48.7°, 50.2°, and 52.4°, respectively, with corresponding crystal planes of (111), (102), (201), (210), (211), (121), (212), (122), (220), (301), (213), and (311). The 2θ positions of low-magnesium calcite diffraction peak were 23.1°, 29.5°, 39.6°, 42.9°, 43.3°, 57.6°, with corresponding crystal planes of (012), (104), (113), (022), (202), (122). With the influence of clay minerals, the intensity of mineral peak induced by *Synechococcus* sp. changed significantly. The strongest d_111_ diffraction peaks of aragonite induced in the control, montmorillonite, and kaolin groups were 102,917, 231,533, and 186,767, respectively. The strongest d_104_ diffraction peaks of low-magnesium calcite were 209,617, 13,517, and 90,533, respectively. These results showed that the intensity of the aragonite diffraction peak increased significantly after the addition of clay minerals, indicating that the crystallinity of aragonite increased with the addition of clay minerals. Meanwhile, the main diffraction peak intensity of low-magnesium calcite decreased significantly with the addition of clay minerals, which was associated with a decrease in crystallinity. As shown in Fig. S2, the low-magnesium calcite content was 37.23%, 13.99%, and 0.24% for the control, montmorillonite, and kaolinite groups, respectively. The aragonite content was 62.77%, 86.01%, and 99.76% for the control, montmorillonite, and kaolinite groups, respectively, indicating that the addition of clay minerals could increase aragonite precipitation.

### Thermogravimetry analysis.

**FIG 4:**
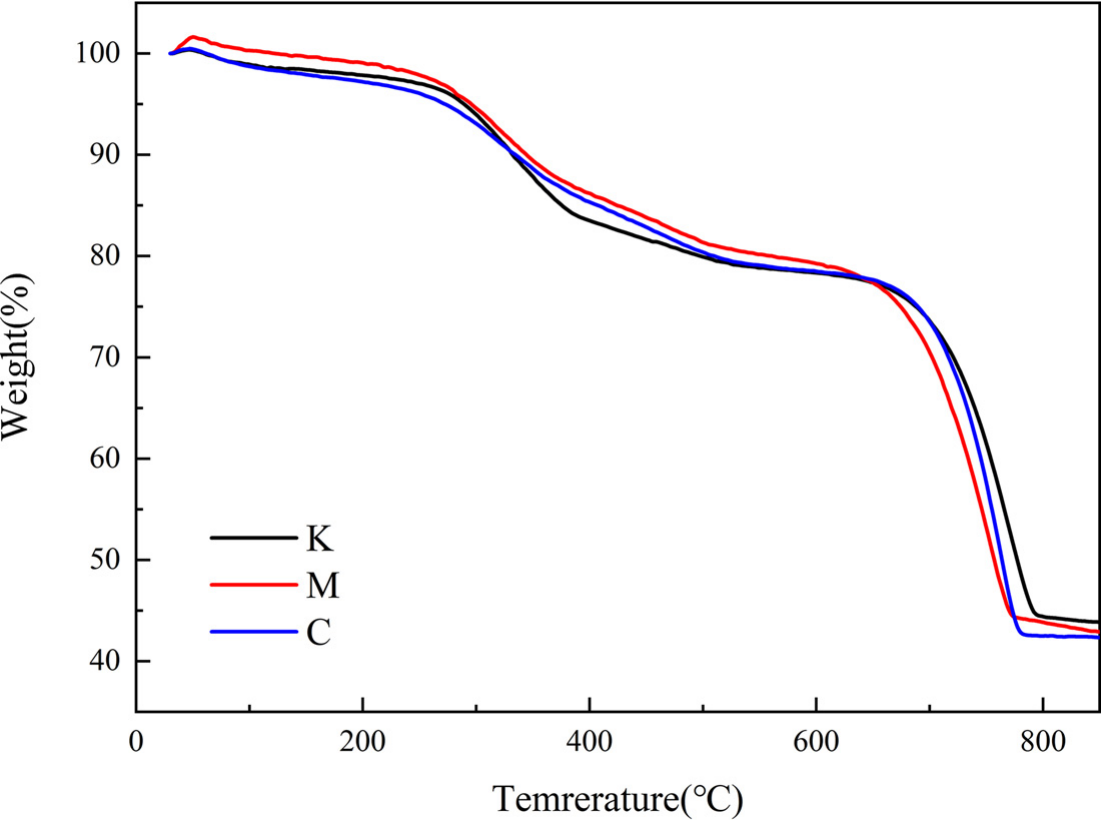
Thermogravimetric sediment analysis.

The organic matter in precipitates were evaluated by thermogravimetry analysis (TGA). The TGA results of the precipitates are shown in Fig. 4. The first stage of weight loss from room temperature to 200°C can be attributed to the water evaporation in the mineral, which indicates that a small amount of free water or crystalline water may be adsorbed on the surface or in sediment pores. The XRD results show that the sample did not contain crystalline water; therefore, the weight loss of this part was free water. The second stage of weight loss from 200°C to 550°C resulted from burned organic matter (22), which indicates that *Synechococcus* sp. and its organic metabolites were reserved in the sediments. The third stage of weight loss from 550°C to 800°C corresponds to the decomposition of CaCO_3_ into CaO and CO_2_ (23). The organic matter loss rates in C, M, and K were 18.11%, 18.92%, and 19.02%, respectively. The montmorillonite and kaolinite group had higher loss rates than the control group, indicating that organic matter could be improved by the aggregation of clay minerals. In other words, clay minerals can accelerate the aggregation of organic matter (*Synechococcus* cells), and kaolinite has a greater aggregation effect on *Synechococcus* cells than montmorillonite.

### Morphology analysis of biogenic carbonates.

Morphology of aggregates was observed by an optical microscope. Figure S3 shows the optical micrographs of the sediments during the carbonates inducing processes. *Synechococcus* cells accumulated at the bottom of the flasks and were surrounded by white translucent flocs. As shown in Fig. S3A, the diameter of the *Synechococcus* cell aggregates was approximately 200 μm. When the clay mineral content increased from 5 to 50 mg/L, the particle size of the aggregates increased from 1,000 μm to between 2,000 and 3,000 μm. When the clay mineral content was 500 mg/L, the *Synechococcus* cells gathered in a band at the bottom. Thus, the aggregate particle size increased with increasing clay mineral content.

**FIG 5:**
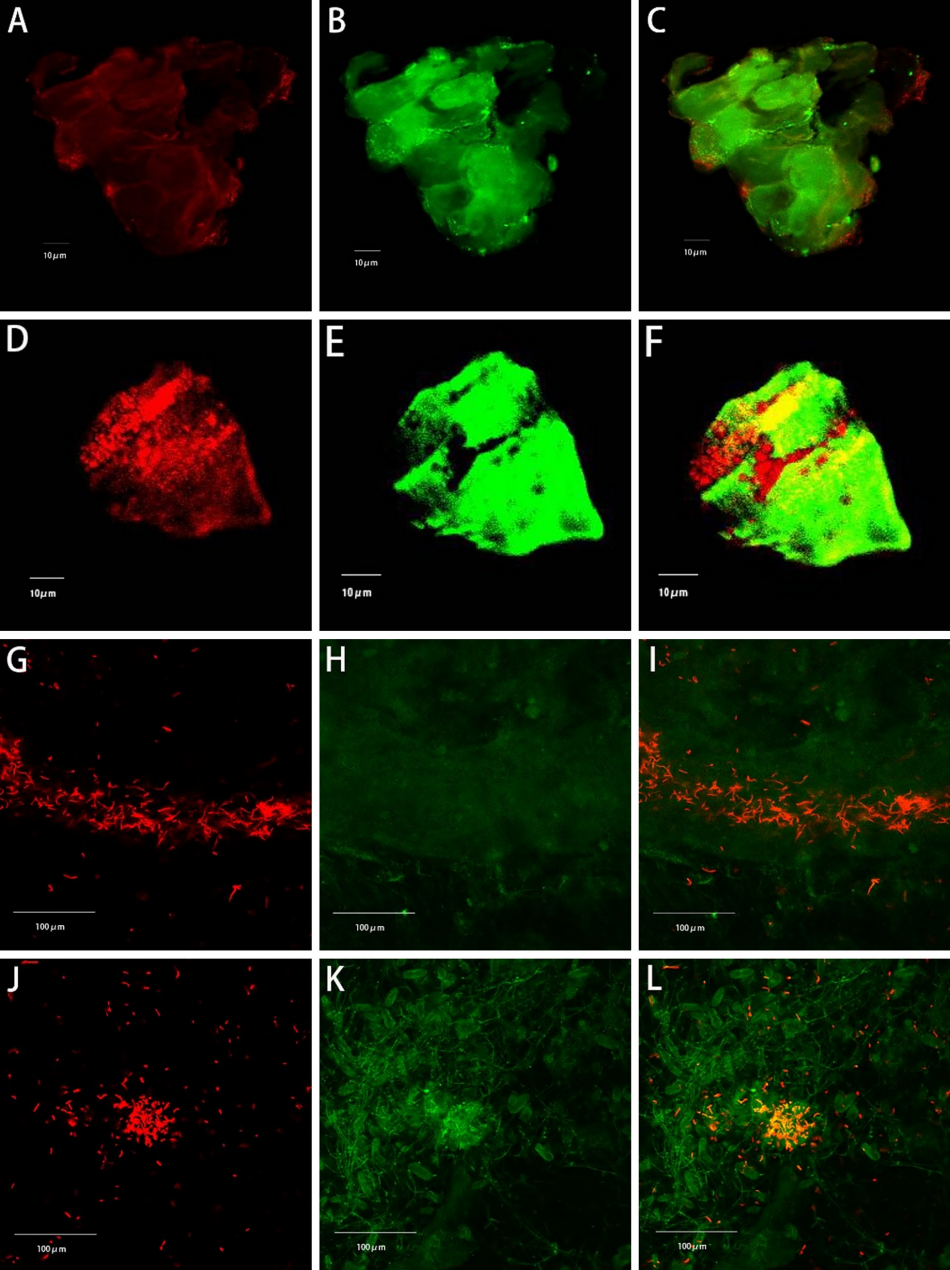
Laser scanning confocal microscope photos. Red is *Synechococcus* cells and green is calcium carbonates stained with calcein.

Carbonates in aggregates were also observed using laser scanning confocal microscopy (LSCM). As shown in Fig. 5, *Synechococcus* cells showed red auto-fluorescence and calcium carbonates showed green fluorescence after staining with calcein AM. Because *Synechococcus* cells are dispersed or embedded in blocky calcium carbonate crystals (Fig. 5A to C), individual cells cannot be distinguished, and only the red fluorescence can be observed. *Synechococcus* cells also aggregated together (Fig. 5D and J), and calcium carbonates precipitated and surrounded the aggregates. Finally, the aggregates were embedded into calcium carbonate crystals with a particle size of approximately 50 μm (Fig. 5D to F). In addition, *Synechococcus* cells were mixed with or surrounded by granular calcium carbonate crystals (Fig. 5L). These aggregates appeared to be orange in color because the fluorescence of cells and calcium carbonates overlapped. The grain size of these carbonates was approximately 15 to 25 μm, which is similar to that of micritic calcite in fine-grained sedimentary rocks.

**FIG 6:**
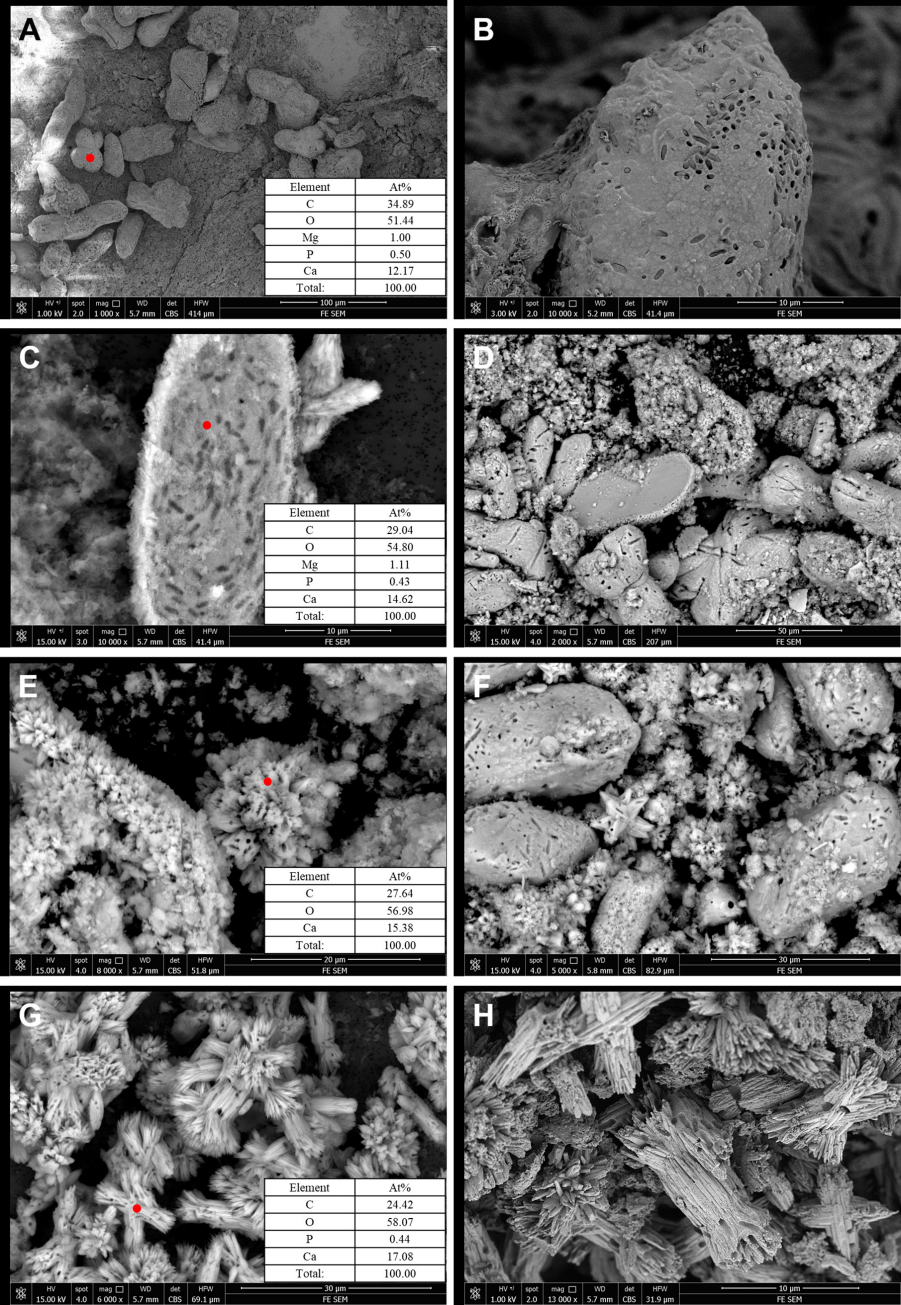
Scanning electron microscopy of carbonates. (A to C) Control group. (D to F) Kaolinite group. (G and H) Montmorillonite group.

Cyanobacteria can induce carbonate around the cyanobacteria cells (24). Organic matter in sediments was removed by H_2_O_2_, the sediment microstructure was observed using scanning electron microscopy (SEM) (Fig. 6). Rod- and clover-shaped carbonate minerals (40 to 70 μm) were observed mainly in the control group (Fig. 6A and B). The energy spectra showed that the rod- and clover-shaped carbonates were low-magnesium calcite. Under a high voltage (15 kV), many holes were observed in the carbonate minerals after H_2_O_2_ oxidation (Fig. 6B and C). The shape and size of these holes were close to those of *Synechococcus* cells, indicating that *Synechococcus* cells aggregated together and carbonate minerals formed around the cells. With the growth of carbonate minerals, *Synechococcus* cells became embedded. However, calcite was larger than micritic calcite in fine-grained carbonate. In addition to the rod-shaped calcite, there were many clustered carbonate minerals in the clay mineral groups (Fig. 6D to H). These carbonate minerals were formed with the addition of clay minerals and were distributed in 5- to 15-μm clusters. Figure 6D to F shows the carbonate minerals of the kaolinite group, and Fig. 6G to H shows the carbonate minerals of the montmorillonite group. Residual holes in *Synechococcus* cells were also observed on the surface and inside the clusters. The cluster mineral was aragonite according to the morphology and energy-dispersive X-ray spectrometer (EDS) analyses. Moreover, aragonite in the montmorillonite group appeared much more compact, with a correspondingly higher crystallinity (Fig. 4). In summary, the control group mainly contained rod-shaped, low-magnesium calcite. In addition to low-magnesium calcite, the kaolinite and montmorillonite groups had many clustered aragonites, and aragonite in the montmorillonite group had a higher crystallinity, which is also consistent with the XRD results. To further determine the origin of carbonates, we analyzed the stable C-isotope composition of the carbonate precipitates. As shown in Table S2, the stable C-isotope values (δ^13^C) were –28.23‰, –22.81‰, and –25.79‰ for the control, montmorillonite group, and kaolinite group, respectively, indicating that the carbonates in precipitates were biogenic (25, 26). Thus, it is evident that *Synechococcus* can induce calcite and aragonite formation under the effect of clay minerals. Compared with kaolinite, montmorillonite had a higher ability to improve the crystallinity of aragonite. Particulate organic carbon could be transported to the ocean depths and sediments via sinking particles (27). The density and sinking velocity of *Synechococcus* aggregates with clay mineral is higher than those of single *Synechococcus* cells, and the sinking velocity can be up to 660 m/d (19). The precipitation of carbonates around *Synechococcus* cells could further increase their density and sinking velocity, and aggregates could protect themselves from consumption by zooplankton or microbes as much as possible (27). The reducing condition of sediments within an anoxic hypolimnion is beneficial for the preservation of organic matters (28). Thus, the formation of aggregates and the precipitation of carbonates are conducive to the sinking and preservation of *Synechococcus*.

## DISCUSSION

### Effect of clay minerals on the aggregation of *Synechococcus*.

Cationic bridges are the main reason for the aggregation of cells on the surface of clay minerals. The points of zero charge (PZC) of kaolinite and montmorillonite are 3.6 and 2.5 (29), respectively. Thus, in a neutral environment, the surfaces of kaolinite and montmorillonite are often negatively charged, and the surface of montmorillonite has a significantly more negative charge than kaolinite. The surface of microorganisms contains negatively charged carboxyl, phosphoryl, amino, and other functional groups, which are also negatively charged (30). Therefore, electrostatic repulsion exists between clay minerals and cyanobacteria cells. Clay minerals and cyanobacteria cells can form cationic bridges through electrolytes, which can overcome the potential energy barrier to achieve adsorption and aggregation (31). Some of the extracellular polysaccharides (EPS) produced by cyanobacteria are composed of uronic acid, an anionic sugar that can form a cationic bridge with negatively charged clay particles (32). Thus, the formation of aggregates depends on the comprehensive force between the clay minerals and the cells. With a lower PZC, the electrostatic repulsion between montmorillonite and *Synechococcus* cells was relatively stronger than that between kaolinite and *Synechococcus* cells, resulting in a relatively lower aggregation rate for montmorillonite (Fig. 1).

### Effect of *Synechococcus* on the Mg/Ca molar ratio.

Clay minerals such as montmorillonite and kaolinite always have large specific surface areas, ion exchange capacities, and adsorption properties (33). However, the concentrations of Ca^2+^ and Mg^2+^ were not significantly affected by the addition of clay minerals. Thus, the reduction in Ca^2+^ or Mg^2+^ concentration was attributed to the effect of *Synechococcus* sp. Carboxyl groups are an important factor for carbonate precipitation. Acidic amino acids such as Glu and Asp are present in the extracellular polymers of cells. Acidic amino acids have two carboxyl groups, one of which is involved in the condensation of proteins, whereas the other is freely retained. Therefore, there is a positive correlation between the number of *Synechococcus* cells and free carboxyl groups. The isoelectric point of the carboxyl group is relatively low (average pK_a_ ≈ 4.7). Most carboxyl groups are deprotonated in neutral or alkaline environments and can bind cations such as Ca^2+^ and Mg^2+^ (34). Because the energy required for the hydration and dehydration of Mg^2+^ is much higher than that for Ca^2+^ (35), Mg^2+^ remained in the culture medium, while much more Ca^2+^ was bound to carboxyl, resulting in an increased Mg^2+^ concentration and Mg/Ca molar ratio (36). Thus, the presence of *Synechococcus* sp. leads to an increase in the Mg/Ca molar ratio.

### Effect of clay minerals on the formation of carbonates.

Mg^2+^ plays an important role in the formation of carbonate minerals. Some researchers have found that the Mg/Ca molar ratio affects the type and composition of carbonate minerals. Therefore, the Mg/Ca molar ratio is regarded as a key kinetic factor in carbonate formation. For example, the mineralization of calcite in the ocean is controlled by the Mg/Ca molar ratio of the surrounding seawater (37), and the magnesium content in carbonate minerals directly affects the type and morphology of minerals (38). It has been reported that calcite morphology is always dumbbell- and cauliflower-shaped in an environment without magnesium (39). With an increasing Mg/Ca molar ratio, bundle- and petal-shaped minerals gradually appear. Mg^2+^ in a high Mg/Ca ratio environment is easily adsorbed on the surface of calcite and further integrates into the crystal structure, inhibiting calcite growth or destroying the crystal structure (40), which results in the formation of aragonites. In other words, the precipitation of calcite crystal nuclei is slowed down, and the crystal nuclei with high magnesium content are redissolved, providing sufficient time for aragonite precipitation. Thus, a low Mg/Ca molar ratio was conducive to the formation of calcite, whereas a high Mg/Ca molar ratio promoted the formation of aragonite. It has also been reported that calcite can be induced when the Mg/Ca ratio is 0, calcite and aragonite are precipitated simultaneously when the Mg/Ca ratio is 2, and only aragonite is induced when the Mg/Ca ratios are 4 and 6 (41). In this study, the Mg/Ca molar ratios were 1.8198 and 1.7206 for the kaolinite and montmorillonite groups, respectively. Clay minerals accelerate the aggregation of *Synechococcus* cells, resulting in increased carboxyl content of aggregates, thus increasing the adsorption of Mg^2+^ and Ca^2+^. Because the carboxyl group has a high affinity for Ca^2+^, we speculate that the Mg/Ca molar ratio of the aggregates’ microenvironment was higher than the measured values, which is favorable for aragonite precipitation. In Fig. 6F, aragonite was coated on the surface of calcite. Therefore, it was inferred that during the early stage of the aggregation process, calcite could be induced by *Synechococcus* cells under a relatively low Mg/Ca ratio. With the growth of aggregates, the Mg/Ca molar ratio increased, resulting in aragonite precipitation. Therefore, clay minerals promote the aggregation of *Synechococcus* cells associated with an increase in the Mg/Ca molar ratio in the surrounding microenvironment, which favors the precipitation of aragonite. It has been reported that carbonates can be precipitated around bacteria cell walls, and bacteria may die and decay shortly after being entombed by these carbonates, leaving many cell-shaped micropores (42). Compared with abiotic carbonates, biotic carbonates in both modern and ancient carbonate crusts have an abundance of micropores (43). Furthermore, the micropores discovered in most of the micritic calcite grains in calcite laminae and fine-carbonate rocks are also induced by cyanobacteria (3, 42). In this study, the negative carbon isotopic values indicate that the carbonates precipitated were biogenic. The aragonite was similar in size (5 to 15 μm) to the micritic calcite of fine-grained sedimentary rocks. Thus, it can be inferred that aragonite can be induced by aggregated cyanobacteria, accompanied by an increase in organic matter, and some aragonite might have evolved into micritic calcite through diagenesis.

## MATERIALS AND METHODS

### Cyanobacterium and culture medium.

The cyanobacterium *Synechococcus* sp. used in this study was purchased from the Institute of Aquatic Biology, Chinese Academy of Sciences. To avoid carbonate precipitation, 200 mL BG11 medium without CO_3_^2–^ and HCO_3_^–^ was prepared in 500-mL Erlenmeyer flasks. The components of the modified BG11 medium were as follows: 1.5 g/L NaNO_3_, 40 mg/LK_2_HPO_4_·3H_2_O, 6 mg/L ferric ammonium citrate, 6 mg/L citric acid, 1 mg/L EDTA, 2.86 g/L H_3_BO_3_, 1.81 g/L MnCl_2_·4H_2_O, 0.222 g/L ZnSO_4_·7H_2_O, 0.079 g/L CuSO_4_·5H_2_O, 0.39 g/L NaMoO_4_·2H_2_O, 0.0494 g/L Co(NO_3_)_2_·6H_2_O, 8.8 g/L Ca(CH_3_COO)_2_·H_2_O, and 10.67 g/L Mg(CH_3_COO)_2_·4H_2_O (44, 45). The pH of the medium was adjusted to 7.0, and then the mixture was autoclaved at 105°C for 30 min. All reagents were of commercial analytical grade. Information about carbonate composition according to the XRD data and thermogravimetric analysis; optical micrographs of sediments in control, kaolinite, and montmorillonite groups, concentrations of Ca^2+^ and Mg^2+^ leached from clay; saturation index, and δ13C values is contained in Supplemental File 1.

### Aggregation of *Synechococcus* cells with clay.

The clay minerals used in this study were kaolinite and montmorillonite (Macklin Inc., China). A batch of kaolinite or montmorillonite with particle sizes of approximately 2.5 μm was added to 200 mL BG11 medium in flasks at concentrations of 5, 50, and 500 mg/L. Next, *Synechococcus* sp. was inoculated. All samples were transferred to a shaker at 130 rpm for 2 h and left to stand for 2 h to allow the aggregates to settle. The absorbance of chlorophyll A of *Synechococcus* in the supernatants was measured to characterize changes in *Synechococcus* cell concentrations. Samples inoculating *Synechococcus* with no clay served as the controls. All samples were in triplicate.

The method for determining chlorophyll A concentration was as follows. The culture medium was centrifuged at 3,000 rpm for 10 min. Then, the supernatant was removed, and the residual was mixed with an equal volume of acetone and incubated at 4°C for 24 h. Next, the supernatant was centrifuged, and the absorbance was measured at 649 and 665 nm using a spectrophotometer. A 90% acetone solution (vol/vol) was used as a blank control. The concentration of chlorophyll A and the aggregation rate were calculated according to the following equations (46, 47):

(1) $$\text{chlorophyll A}=12.47A_{665}-3.62A_{649}$$

(2) $$\text{aggregation rate }=\frac{{\text{chlorophyll A}}_{\;\mathrm{Synechococcus}\;\text{only}}-{\text{ chlorophyll A}}_{\text{ clay group}}}{{\text{chlorophyll A}}_{\;\mathrm{Synechococcus}\text{ only}}}\times 100\%$$

### Carbonate-inducing experiment.

Kaolinite and montmorillonite were added separately to 200 mL BG11 medium. The clay concentrations were 0 and 50 mg/L. Afterwards, *Synechococcus* sp. was inoculated in BG11 medium and cultured in a rotating shaker at 130 rpm at 25°C. The light intensity was 2,000 LUX and 12:12-h light-dark cycles were applied for 12 days. All samples were in triplicate.

### Chemical analysis.

The samples described in the section “carbonate-inducing experiment” were used to measure the pH, stable carbon isotope value (δ^13^C), and concentrations of Mg^2+^ and Ca^2+^. The pH of the cultures was measured using a pH meter (PHS-3C; Thunder Magnetic, China) at 48-h intervals. Meanwhile, to measure the adsorption of Mg^2+^ and Ca^2+^, kaolinite and montmorillonite were added to BG11 medium without *Synechococcus*. In addition, kaolinite and montmorillonite were added to deionized water instead of BG11 medium to evaluate the Mg^2+^ and Ca^2+^ leached from clay minerals. The Mg^2+^ and Ca^2+^ concentrations were measured after 12 days of cultivation using an inductively coupled plasma-optical emission spectrometer (ICP-OES, Perkin Elmer 8300, Germany), and the ratio of magnesium to calcium was calculated. Stable carbon isotopic analysis was performed using a stable isotope mass spectrometer (MAT253, Thermo Fisher Scientific, USA). The saturation index of calcite and aragonite after incubation by the geochemical modeling program PHREEQC (48). All samples were analyzed in triplicate.

### Sediment composition and morphology observations.

The precipitated minerals were collected and purified and examined by XRD (D/Max-RC, Japan), TGA (Mettler-Toledo TGA/DSC1/1600LF, Switzerland), and Fourier transform infrared (FT-IR) spectroscopy (VERTEX 70 FTIR, Germany).

The morphology of the minerals was observed using a laser confocal microscope (FluoView FV1000, Japan) and SEM (FEI NanoSEM 450, USA) coupled with an energy-dispersive X-ray spectrometer (Oxford X-max^50^, Oxford, United Kingdom). For laser confocal microscopy, calcein was used to dye carbonate minerals (49). The precipitates in the culture medium were then collected and centrifuged at 1,660 rpm for 10 min, washed three times with phosphate-buffered saline (PBS) buffer, soaked and fixed with 4% (vol/vol) formalin solution, and stained with calcein (1 μg/L) for 2 h at 25°C (24). The excitation wavelength was 490 nm, and the emission wavelength was 505 to 560 nm. The calcein stain did not bind to clay (data not shown). *Synechococcus* cells were identified by the autofluorescence of chlorophyll A in the far-red channel (excitation, 633 nm; emission, 650 to 800 nm). For the SEM method, one portion of the precipitate was soaked in 10% H_2_O_2_ (vol/vol) to remove organic matter, washed with distilled water, and dried at 70°C for 10 h. Another portion of the precipitate was soaked in glutaraldehyde and dried at the critical point of carbon dioxide. The minerals were then observed by SEM at 5 to 15 kV.

### Conclusion.

Clay minerals can promote the aggregation of *Synechococcus* cells. The aggregating particle size increased with increasing clay mineral content, resulting in increased organic matter content in the sediments, and the aggregation effect of kaolinite was significantly stronger than that of montmorillonite. Low-magnesium calcite mainly precipitated by the induction of *Synechococcus* sp., the aggregation of *Synechococcus* sp. increased the carboxyl group content in aggregates, which further improved the Mg/Ca molar ratio in the aggregates’ microenvironment, resulting in much more aragonite precipitation. Thus, the aggregation of *Synechococcus* by clay minerals accelerated the precipitation of aragonite (5 to 10 μm), inferring that some micritic calcite in oil shale may have originated from the diagenesis of aragonite induced by cyanobacteria under the effect of clay minerals.
