# Supplementary material for: Effect of Clay Minerals on Carbonate Precipitation Induced by Cyanobacterium Synechococcus sp
Source: Microbiol Spectr. 2023 Apr 11;11(3):e00363-23. doi: 10.1128/spectrum.00363-23 (PMC10269649; doi:10.1128/spectrum.00363-23)
Supplement: Supplemental file 1 — Supplemental material. Download spectrum.00363-23-s0001.pdf, PDF file, 0.6 MB [file spectrum.00363-23-s0001.pdf]

Effect of clay minerals on carbonate precipitation induced by cyanobacterium *Synechococcus* sp.

Xiao Wang[a], Xiangxin Kong[b], Qian Liu[a], Kun Li[a], Zaixing Jiang[b], Hengjun Gai[a], Meng Xiao[a*]

[a] State Key Laboratory Base for Eco-Chemical Engineering in College of Chemical Engineering, Qingdao University of Science and Technology, Qingdao 266042, China

[b] School of Energy Resources, China University of Geosciences (Beijing), Beijing 100083, China

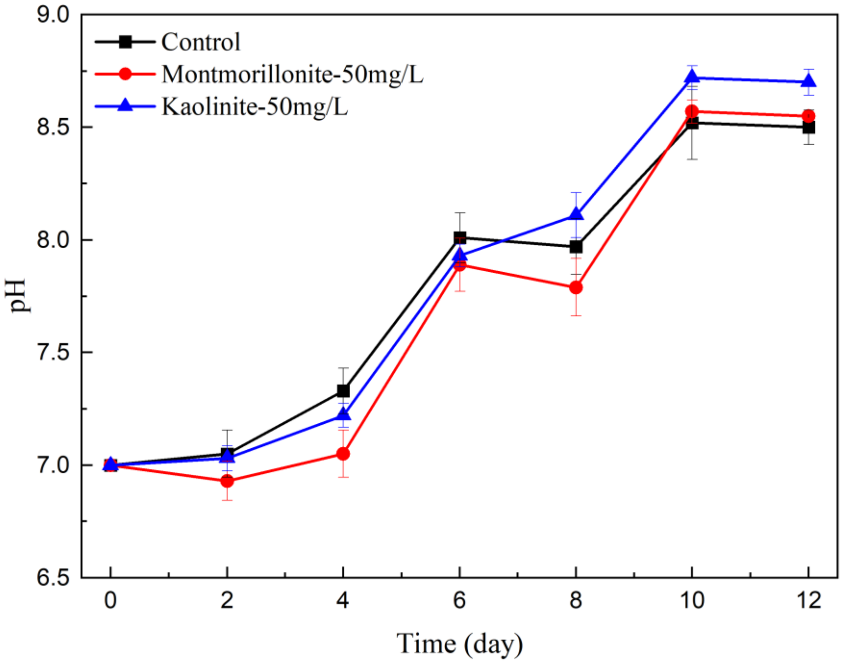

Figure S1. Changes of pH value with time for different samples

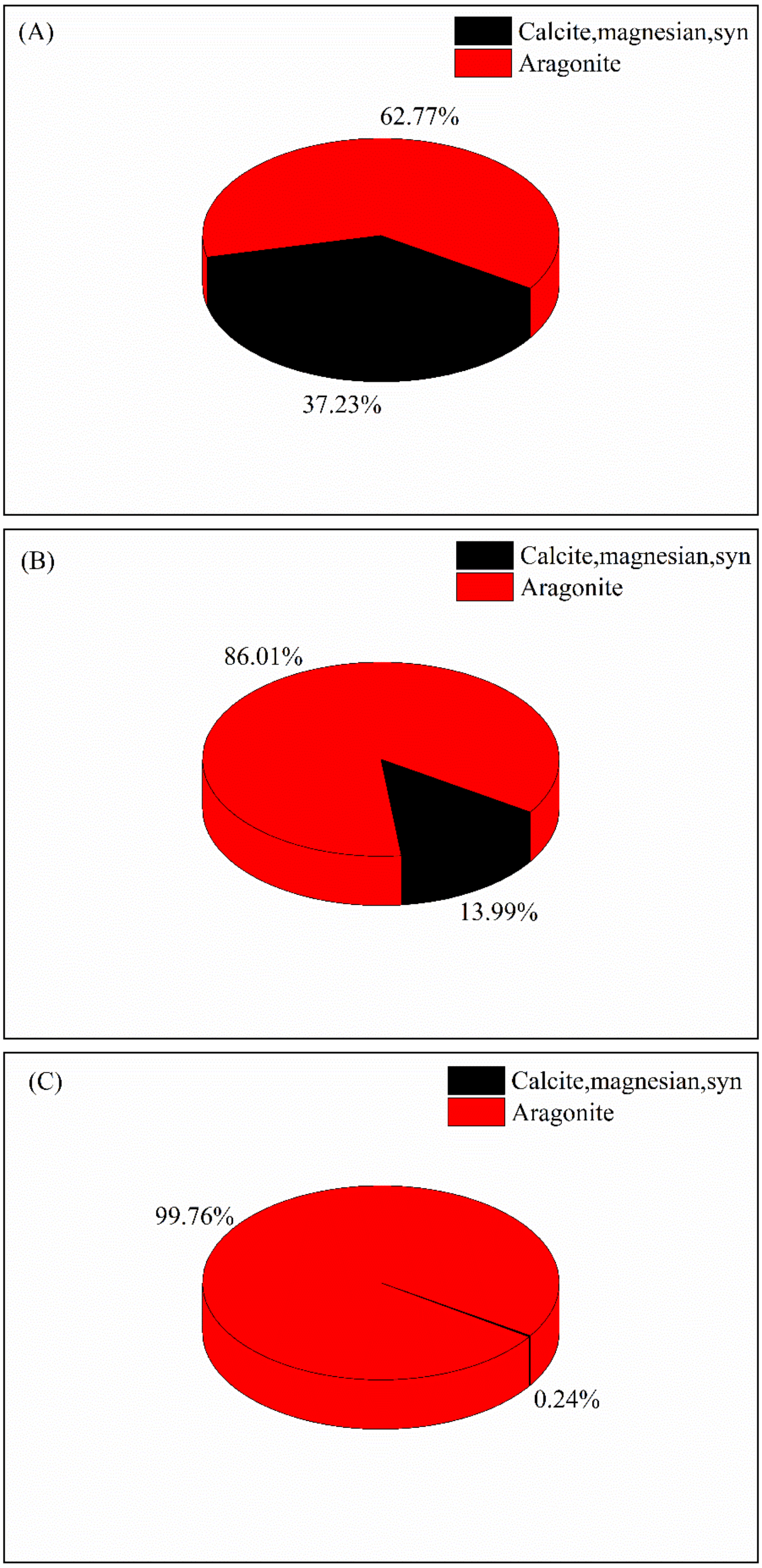

Figure S2. Carbonates composition according to the XRD data. (A) Control group; (B) Montmorillonite group; (C) Kaolinite group

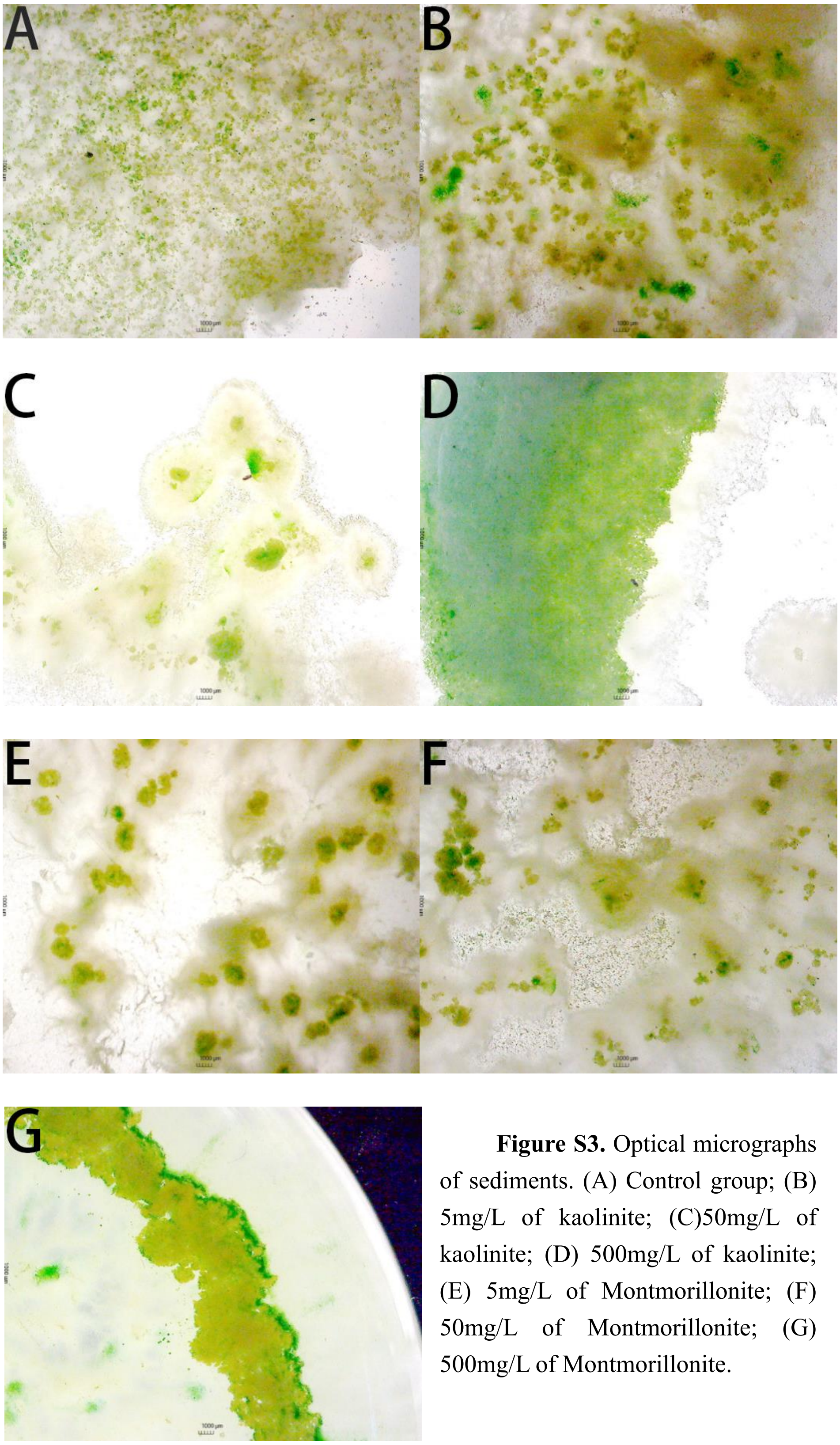

Figure S3. Optical micrographs of sediments. (A) Control group; (B) 5mg/L of kaolinite; (C)50mg/L of kaolinite; (D) 500mg/L of kaolinite; (E) 5mg/L of Montmorillonite; (F) 50mg/L of Montmorillonite; (G) 500mg/L of Montmorillonite.

Table S1 The concentration of Ca^{2+} and Mg^{2+} leached from clay (μg/L)

	Ca^{2+}	Mg^{2+}
K-50	87.95±0.012	41.78±0.061
M-50	69.42±0.261	32.31±0.001

Table S2 Saturation index and $\delta^{13}C$ values

Group	Saturation index		$\delta^{13}C$ (‰)
	Aragonite	Calcite	
Control	1.717	1.861	-28.23±0.068
M-50	1.74	1.884	-22.81±0.073
K-50	1.754	1.898	-25.79±0.057
